# New York

**Published:** 1896-09

**Authors:** 


*New York.* The failure to make an appropriation to carry on the work of cattle inspection in the Empire State has practically halted this great commonwealth in the good work, and we are sure that the sharp criticisms at home and from contiguous States will awaken the lawmakers to a better sense of duty at the next session.

